# Supplementary material for: Multi-petahertz electron interference in Cr:Al2O3 solid-state material
Source: Nat Commun. 2018 Apr 18;9:1468. doi: 10.1038/s41467-018-03885-7 (PMC5906618; doi:10.1038/s41467-018-03885-7)
Supplement: Supplementary file 1 — Supplementary Information [file 41467_2018_3885_MOESM1_ESM.pdf]

Supplementary Information

Multi-petahertz electron interference in $Cr:Al_2O_3$ solid-state material

Hiroki Mashiko[1], Yuta Chisuga[1,2], Ikufumi Katayama[2], Katsuya Oguri[1], Hiroyuki Masuda[1,2], Jun Takeda[2], and Hideki Gotoh[1]

[1]NTT Basic Research Laboratories, 3-1 Morinosato Wakamiya, Atsugi, Kanagawa 243-0198, Japan. [2] Department of Physics, Graduate School of Engineering, Yokohama National University, 79-5 Tokiwadai, Hodogaya, Yokohama 240-8501, Japan. Correspondence and requests for materials should be addressed to H.M. (mashiko.hiroki@lab.ntt.co.jp)

Supplementary note 1: Experimental setup

A few-cycle near-infrared (NIR) pulse (1.55-eV centre photon energy with 7-fs duration) from a Ti:sapphire laser was used for the pump-NIR pulse in the Fourier transform extreme ultraviolet attosecond spectroscopy (FTXUV) based on transient absorption. The NIR pulse is also used to generate the isolated attosecond pulse (IAP) via the double optical gating (DOG) method[1]. The IAP is used as the probe-pulse in the FTXUV method. The pump-and-probe system is described in refs. (2) and (3). The stability of the pump-probe system is 23-as timing jitter at the root mean square over 12 hours, which is monitored by a co-propagated continuous-wave laser (633-nm wavelength)[2]. The target intensity of the pump-NIR pulse is approximately 2×10^{12} W/cm^2, which is estimated from the photoelectron energy shift with the intensity dependence of the attosecond streak[4]. The collinearly propagated IAP and NIR pulse are focused onto the target of alumina with chromium dopant (Cr:Al_2O_3). After the target, the transmitted IAP is sent to an extreme ultraviolet (XUV) spectrometer equipped with a micro-channel plate and a cooled charge-coupled device camera. The spectral resolution is 120 meV at 45.5-eV photon energy[3].

Supplementary note 2: Temporal characterisation of IAP

To confirm the IAP duration, we use the frequency-resolved optical gating for complete reconstruction of attosecond bursts (FROG-CRAB) method[5] based on an attosecond streak camera[4]. The collinearly propagated IAP and NIR pulse are focused to the gas jet with helium atom (50-μm interaction length; 740-mbar backing pressure). The estimated target intensity of the NIR pulse is approximately 2×10^{12} W/cm^2 in this measurement. The ionized photoelectrons induced by the IAP are detected by a regular time-of-flight system. Supplementary Figures 1(a) and (b) show the experimental and retrieved FROG-CRAB traces. Supplementary Figure 1(c) shows the reconstructed temporal profile and phase of the IAP pulse. The duration is 192 as at the full width at half maximum (FWHM). The IAP spectrum reconstructed by the FROG-CRAB method (red solid line) agrees well with the measured spectrum (blue dashed line), as shown in Supplementary Figure 1(d).

Supplementary note 3: Cr:Al_2O_3 target

In this experiment, trigonal (rhombohedral) α-Al_2O_3 is used for the host material, which has the band-gap energy of 8.7 eV[6]. The α-Al_2O_3 is doped with the Cr material during the single-crystalline α-Al_2O_3 crystal growth. The Cr^{3+} ions produce a donor-like intermediate level for the Al_2O_3 host material. Supplementary Figure 2 shows the atomic number densities of the Cr dopant and the Al_2O_3 host material. The density of Al_2O_3 is 2×10^{22} cm^{-3}. The density of Cr is estimated to be 2×10^{17} cm^{-3} by secondary ion mass spectrometry (SIMS), which gives rise to the doping level of approximately 1×10^{-3} at. % (10 ppm).

The thin 36-nm-thick target without a substrate was manufactured from a 400-μm-thick bulk target by NTT-AT Inc.[7] by mechanical polishing and ion beam milling. Commonly, the technology is used for the process in transmission electron microscopy. The target has thickness graduation from a few nanometers to a few hundred micrometers. It was mounted on ring holder equipped with a linear electronic actuator to select the proper thickness.

Supplementary Figure 3 shows the optical density (OD) using the IAP without the NIR pulse. Here, we defined the OD at laser frequency ω as $\mathrm{OD}(\omega)=\log[I_{\mathrm{IAPin}}(\omega)/I_{\mathrm{IAPout}}(\omega)]$, where $I_{\mathrm{IAPin}}(\omega)$ is the spectrum of the input IAP. The $I_{\mathrm{IAPout}}(\omega)$ is the absorption spectrum with the target, and it also corresponds to the transmitted spectrum from it. The OD(ω) is proportional to the regular absorption coefficient. Consequently, the OD(ω) monitors the spectral deviation with the target. The effective thickness of the target is 36 nm, which is

directly estimated from the IAP absorption using the extinction coefficient from ref. (8). The target was used for the transient absorption spectroscopy with the NIR pulse.

Supplementary note 4: Simulation of resonant polarizations

To calculate the resonant high-order polarizations in the Cr:Al_2O_3, we simply consider the time-dependent density matrix formalism with a two level system[9,10] whose energy separation is either resonant to the donor-like state corresponding to the fifth-order component ($5\hbar\omega$) or resonant to the conduction band (CB) corresponding to the seventh-order component ($7\hbar\omega$). The Hamiltonian system is expressed as in Ref. (10):

$$H = H_0 + \boldsymbol{\mu} \cdot \mathbf{F} = \begin{pmatrix} E_0 & \mu F \\ \mu^* F & E_1 \end{pmatrix}. \quad (1)$$

Here, $\boldsymbol{\mu}$ and μ are the dipole moment and its magnitude projected along the applied electric laser field, respectively. $\mathbf{F}$ and F are the field vector and its strength of the applied laser pulse, which is given by $F(t) = Ae^{-t^2/2\sigma}\sin(\omega t)$, where A is the field amplitude, 2σ (=7 fs) is the pulse duration, and ω (=2π×0.375 PHz) is the centre angler frequency of the laser pulses. By numerically solving

$$\frac{\partial\rho}{\partial t} = -\frac{i}{\hbar}[H,\rho] - \frac{1}{\tau}\begin{pmatrix} 0 & \rho_{01} \\ \rho_{10} & 0 \end{pmatrix} \quad (2)$$

with a phenomenological dephasing time τ, we could obtain the time-dependent matrix elements ρ_{ij} (i, j=0, 1) of the density matrix ρ and the polarization P=Tr($\mu\rho$). Here, the longitudinal relaxation is neglected, the initial state of the system was assumed to be the ground state, and the matrix element of the dipole moment $\boldsymbol{\mu}$ was assumed to be a real number.

The calculated results with the parameter value of $\mu F/\hbar\omega$=0.59 are shown in Fig. 4 in the main text. In the simulation, we used τ_{donor}=3 fs for the donor-like state and τ_{CB}=0.2 fs for the CB state. The result corresponds to the NIR-field-induced polarization under the perturbative regime, where the polarization amplitude decreases with the increasing order of the harmonics. The resonant polarization can be extracted by Fourier filtering, and the inverse Fourier transform of the filtered polarization gives the time-domain waveform as shown in Fig. 4(b) in the manuscript. Here, if the dephasing time is shorter than the pulse duration of the NIR pulse, the polarization almost follows the harmonics of the NIR electric field. On the contrary, if the dephasing time is much longer than the pulse duration, the resonant polarization builds up until the NIR pulse passes by. Therefore, the peak position of the time-domain polarization will be delayed if the dephasing time is long compared with the pulse duration. This is exactly what we observed in the experiment, where the low-order harmonics (4th and 5th) are delayed by 2 fs compared with the high-order harmonics (6th and 7th).

The measured relative time delay of approximately 2 fs, as shown in Fig. 3, could originate from the difference in an intra-band electron-electron scattering in the donor-like and CB states. Generally, the dephasing time in the spatially localized energy state is much longer than that in the band energy state[11,12]. Thus, the spatially localized Cr donor-like state with the low doping level has many fewer relaxation channels in the unoccupied state compared with the CB state. In addition, the dephasing time is commonly explained by the density- and energy-dependent damping rate, which is proportional to cube root of the excited carrier-density[13] n. The value of n is given by

$$n = \frac{\alpha(1-R)P}{\pi w_0^2 v\hbar\omega}, \quad (3)$$

where $\hbar\omega$ is the photon energy of the pump pulse and the v is the repetition rate of laser[14]. The w_0 is beam spot size. The R and α are the reflectivity at normal incidence and the linear

absorption coefficient, respectively. The term of $P/(\nu\pi w_0^2)$ corresponds to the incident pump fluence. Here, the estimated fluence of the NIR pulse is 7 mJ/cm^2 on the target. The reflectivity R is from ref. (8). Here, we use the linear absorption coefficient α, as shown in Fig. 2(c), because each nonlinear absorption coefficient of the 4-7th orders is difficult to directly determined in the Cr:Al_2O_3 target. The estimated excited carrier densities are $n_{4\hbar\omega}$=5.5×10^{16} cm^{-3} (6.2 eV), $n_{5\hbar\omega}$=5.2×10^{16} cm^{-3} (7.7 eV), $n_{6\hbar\omega}$=1.8×10^{21} cm^{-3} (9.2 eV), and $n_{7\hbar\omega}$=2.6×10^{21} cm^{-3} (10.8 eV). Actually, the values should be much lower with a perturbative multiphoton process. However, the largely different carrier densities could produce the individual dephasing times in the donor-like and CB states. Thanks to the reduced pulse duration of the high-order harmonics as well as the sub-cycle time-resolution of our setup, we could clearly visualize the ultrafast time delay due to the ultrafast dephasing. Consequently, even using the simple time-dependent density matrix formalism, we could reproduce the remarkable characteristics of the observed multi-petahertz polarizations in the Cr:Al_2O_3.

Supplementary References

1. Mashiko, H. *et al.* Double optical gating of high-order harmonic generation with carrier-envelope phase stabilized lasers. *Phys. Rev. Lett.* **100,** 103906 (2008).
2. Mashiko, H., Oguri, K., Yamaguchi, T., Suda, A., and Gotoh, H. Petahertz optical drive with wide-bandgap semiconductor. *Nat. Phys.* **12,** 741-745 (2016).
3. Mashiko, H., Yamaguchi, T., Oguri, K., Suda, A., and Gotoh, H. Characterizing inner-shell with spectral phase interferometry for direct electric-field reconstruction. *Nat. Commun.* **5,** 5599 (2014).
4. Itatani, J. *et al.* Attosecond streak camera. *Phys. Rev. Lett.* **88,** 173903 (2002).
5. Mairesse Y., and Quéré, F. Frequency-resolved optical gating for complete reconstruction of attosecond bursts. *Phys. Rev. A* **71,** 011401(R) (2005).
6. Dobrovinskaya, E. R., Lytvynov, L. A., and Pishchik, V. *Sapphire: material, manufacturing, applications*. (Springer Science & Business Media, 2009).
7. http://www.ntt-at.com/
8. French, R. H., Müllejans, H., and Jones, D. J. Optical properties of aluminum oxide: determined from vacuum ultraviolet and electron energy-loss spectroscopies. *J. Am. Ceram. Soc.* **81,** 2549–2557 (1998).
9. Junginger, F. *et al.* Nonperturbative interband response of a bulk InSb semiconductor driven off resonantly by terahertz electromagnetic few-cycle pulses. *Phys. Rev. Lett.* **109,** 147403 (2012).
10. Casperson, L. W. Few-cycle pulses in two-level media. *Phys. Rev. A* **57,** 609-621 (1998).
11. Webb, M. D., Cundiff, S. T., and Steel, D. G. Stimulated-picosecond-photon-echo studies of localized exciton relaxation and dephasing in GaAs/AlxGa1-xAs multiple quantum wells. *Phys. Rev. B* **43,** 12658-12661 (1991).
12. Oohashi, H., Ando, H., and Kanbe, H. Homogeneous linewidth of bound excitons in high-purity GaAs measured by spectral hole burning. *Phys. Rev. B* **54,** 4702-4706 (1996).
13. Vu, Q. T. *et al.* Light-induced gaps in semiconductor band-to-band transitions. *Phys. Rev. Lett.* **92,** 217403 (2004).
14. Letcher, J. J., Kang, K., Cahill, D. G., and Dlott, D. D. Effects of high carrier densities on phonon and carrier lifetimes in Si by time-resolved anti-Stokes Raman scattering. *Appl. Phys. Lett.* **90,** 252104 (2007).

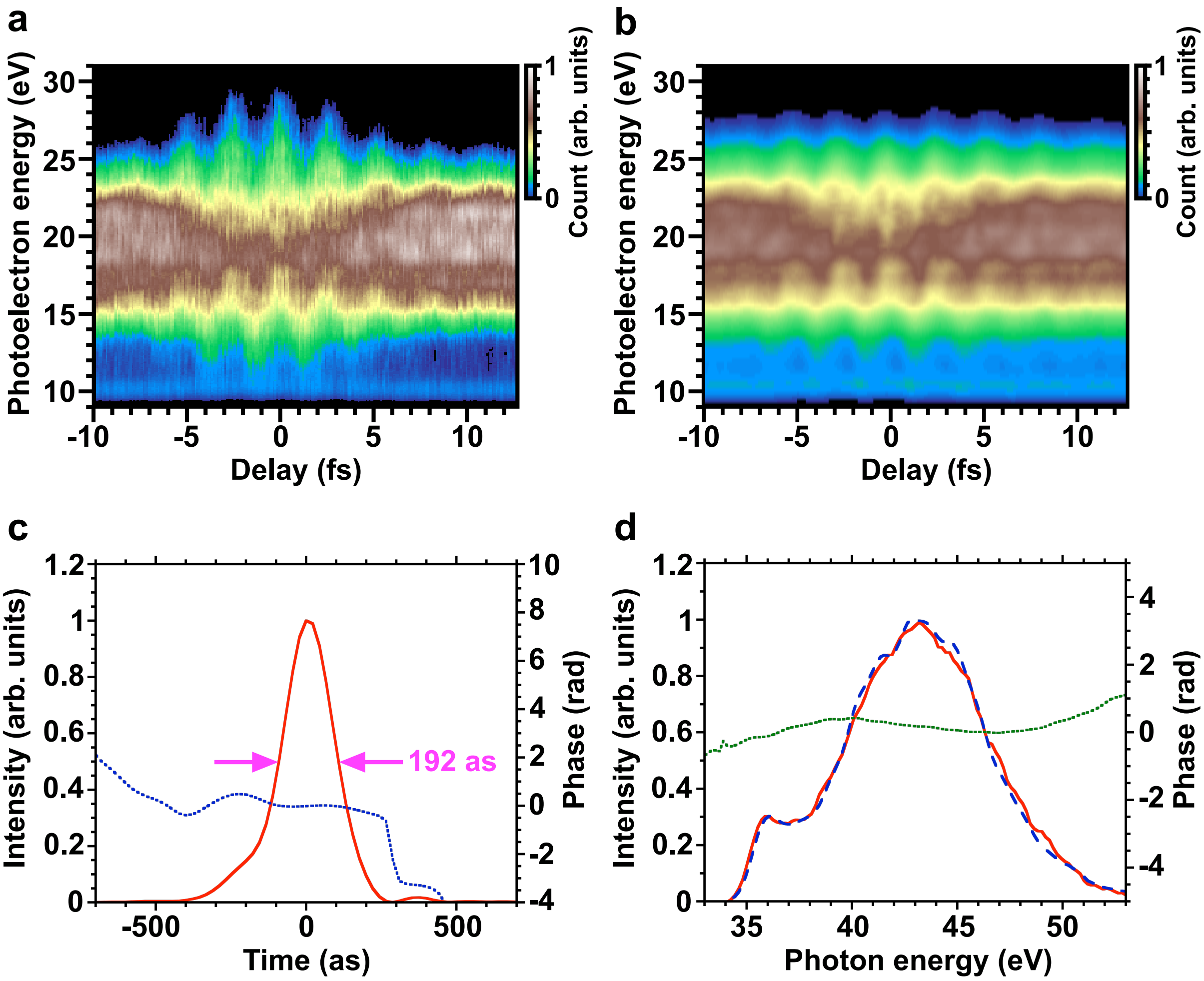

Supplementary Figure 1| Temporal characterisation of IAP. (**a**) Measured and (**b**) reconstructed FROG-CRAB traces using helium atoms. (**c**) Reconstructed temporal profile (red solid line) and phase (blue dotted line). The duration is 192 as at the FWHM (pink arrow). (**d**) Reconstructed spectrum (red solid line) and phase (green dotted line). For comparison, the measured spectrum (blue dashed line) without the streak field of the NIR pulse is also shown.

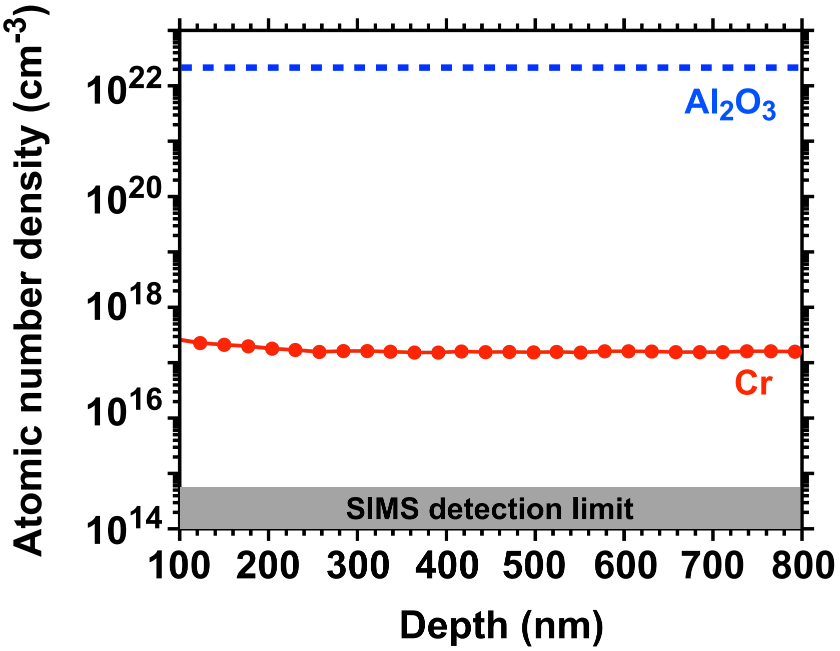

Supplementary Figure 2| Atomic number densities of the Cr dopant and the Al_2O_3 host material. The density of Al_2O_3 is 2×10^{22} cm^{-3} (blue dashed line). The density of Cr (red filled-circle solid line), directly measured by the secondary ion mass spectrometry (SIMS), is 1×10^{17} cm^{-3}. The estimated doping level of Cr is approximately 1×10^{-3} at. % (10 ppm). The black shaded area shows the detection limit of SIMS.

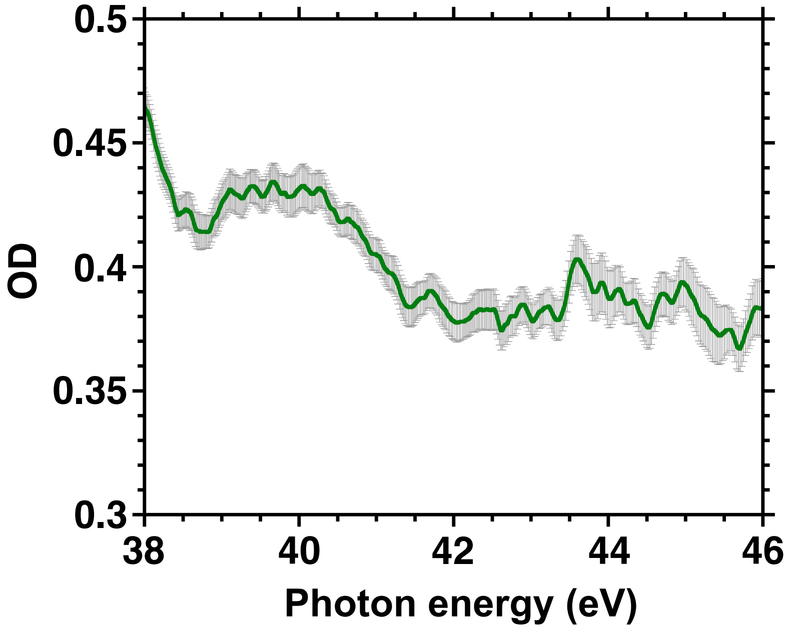

Supplementary Figure 3| Measured optical density (OD) with Cr:Al_2O_3 using the IAP. The OD value is proportional to the regular absorption coefficient. The error bar represents the standard deviation in ten measurements.
